# Molecular Epidemiology of Influenza A(H1N1)pdm09 Virus among Humans and Swine, Sri Lanka

**DOI:** 10.3201/eid2012.140842

**Published:** 2014-12

**Authors:** Harsha K.K. Perera, Dhanasekaran Vijaykrishna, Akuratiya G. Premarathna, Chrishan J.S. Jayamaha, Geethani Wickramasinghe, Chung L. Cheung, Ming F. Yeung, Leo L.M. Poon, Aluthgama K.C. Perera, Ian G. Barr, Yi Guan, Malik Peiris

**Affiliations:** University of Hong Kong, Hong Kong, China (H.K.K. Perera, C.L. Cheung, M.F. Yeung. L.L.M. Poon, Y. Guan, M. Peiris);; University of Kelaniya, Kelaniya, Sri Lanka (H.K.K. Perera, A.G. Premarathna);; Duke-National University of Singapore Graduate Medical School, Singapore (D. Vijaykrishna);; Medical Research Institute, Colombo, Sri Lanka (C.J.S. Jayamaha, G. Wickramasinghe);; Colombo Municipal Council, Colombo (A.K.-C. Perera);; WHO Collaborating Centre for Reference and Research on Influenza, Melbourne, Australia (I.G.Barr)

**Keywords:** Influenza, viruses, A(H1N1), pandemic, pdm09, 2009, biosecurity, evolution, interspecies transmission, human, swine, fomite, Sri Lanka

## Abstract

After multiple discrete introductions of influenza A(H1N1)pdm09 virus into Sri Lanka, the virus was transmitted among humans, then swine. The spread of virus between geographically distant swine farms is consistent with virus dispersal associated with a vehicle used for swine transportation, although this remains unproven.

The first known transmission of influenza A(H1N1)pdm09 virus to humans from swine was in 2009. As the virus spread among humans worldwide, it was transmitted from humans to swine repeatedly ( *1* ), changing the global genetic landscape of swine influenza viruses. We previously reported the spillover of H1N1pdm from humans to swine and absence of North American triple reassortant, classical, and European avian-like swine viruses in swine herds in Sri Lanka during August 2009�?"May 2012 ( *2* ). Here, we extend these studies through August 2013 with the analysis of paired nasal and tracheal swab samples collected from 4,683 animals and serum samples from 3,351 animals ( Technical Appendix Table 1) and comprehensively analyze full genomes of viruses isolated from samples from 26 swine (11 isolated in 2009, 4 in 2010, and 11 in 2011) and 35 humans (6 isolated in 2009, 17 in 2010, 9 in 2011, and 3 in 2012) and 2 publicly available hemagglutinin sequences of human H1N1pdm viruses from Sri Lanka. Sequences generated in this study are available in GenBank (KJ856002�?"KJ856446). 

To understand the molecular epidemiology and spatial and temporal dynamics of spillover events, we compared our data with full-genome sequences of H1N1pdm available in public databases as of August 28, 2013. These include all available full genome sequences from swine H1N1pdm viruses (n = 82), all human H1N1pdm viruses from outside of the USA (n = 957), and 100 randomly selected full genome sequences from 1,500 human H1N1pdm sequences from the United States. Reassorted swine or human viruses containing H1N1pdm virus genes were excluded from this analysis. Our final dataset included the full genomes of 35 human and 26 swine samples from Sri Lanka and a global sample of 1,057 human and 82 swine virus sequences.

The single breakpoint recombination and genetic algorithm for recombination detection methods ( *3* ) excluded the presence of reassortants in our dataset; hence, we used concatenated genomes of 8 gene segments for all subsequent analyses. We conducted multiple-sequence alignment using MUSCLE ( *4* ) and optimized the sum of all of the pairs of characters manually. Phylogenetic trees and bootstrap supports were estimated by using the GTR+I+I" nucleotide substitution model as identified by using JModeltest ( *5* ) and the maximum likelihood method in RaXML ( *6* ). We inferred dates of introduction of major Sri Lankan human and swine lineages using the relaxed clock method under a Bayesian Markov chain Monte Carlo approach (BEAST v1.7) ( *7* ).

Figure 1 illustrates independent introductions of at least 8 H1N1pdm sublineages into Sri Lanka during 2009�?"2012. Six of these were exclusively detected in humans: hu1 (2009), hu2 (2009), hu3 (2009), hu5 (2010/11), hu6 (2011), and hu7 (2011). One was exclusively from swine (sw1; 2009/10) and 1 sublineage was detected in both humans (hu4; 2011) and swine (sw2; 2011) ( Figures 1 , 2 ). Similar multiple discrete introductions of human H1N1pdm viruses have been reported in the United Kingdom and India ( *8* , *9* ).

**Figure 1:**
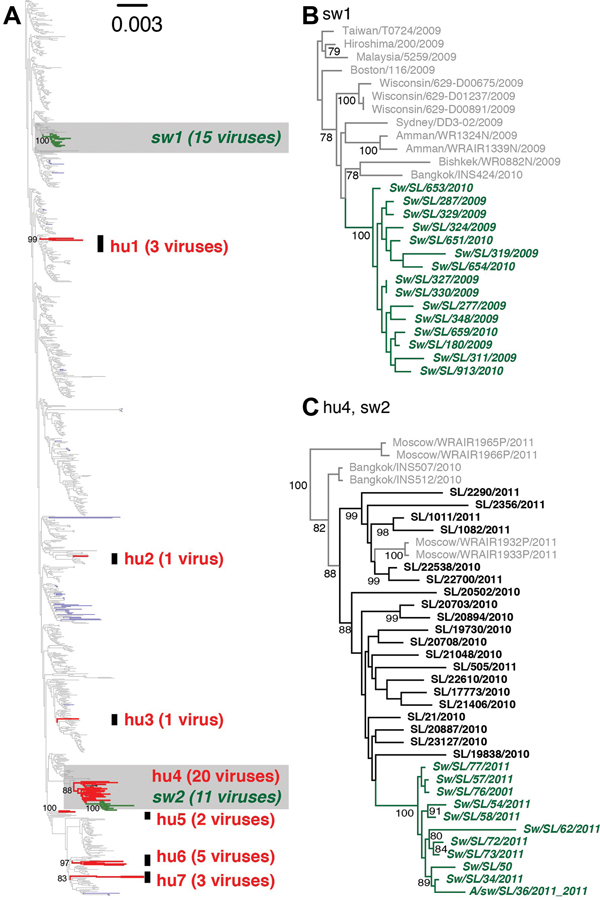
Phylogenetic relationships of influenza A H1N1pdm viruses isolated from human and swine during 2009�?"2012 in Sri Lanka. (A) Maximum likelihood tree generated from a concatenated dataset of 8 gene segment sequences from 1,057 human and 82 swine H1N1pdm viruses isolated globally during 2009�?"2012, and 35 human and 26 swine H1N1pdm viruses isolated in Sri Lanka in 2009�?"2012. Red and green branches represent human (hu) and swine (sw) viruses isolated in Sri Lanka, respectively; gray and blue branches represent globally sampled human and swine viruses, respectively. Highlighted regions (gray) are shown with virus names in (B) and (C), respectively. Support values estimated from 500 maximum likelihood bootstrap replicates are shown along the node for each swine and human clusters identified in Sri Lanka in (A) and for each node with >70% support for (B) and (C).

**Figure 2:**
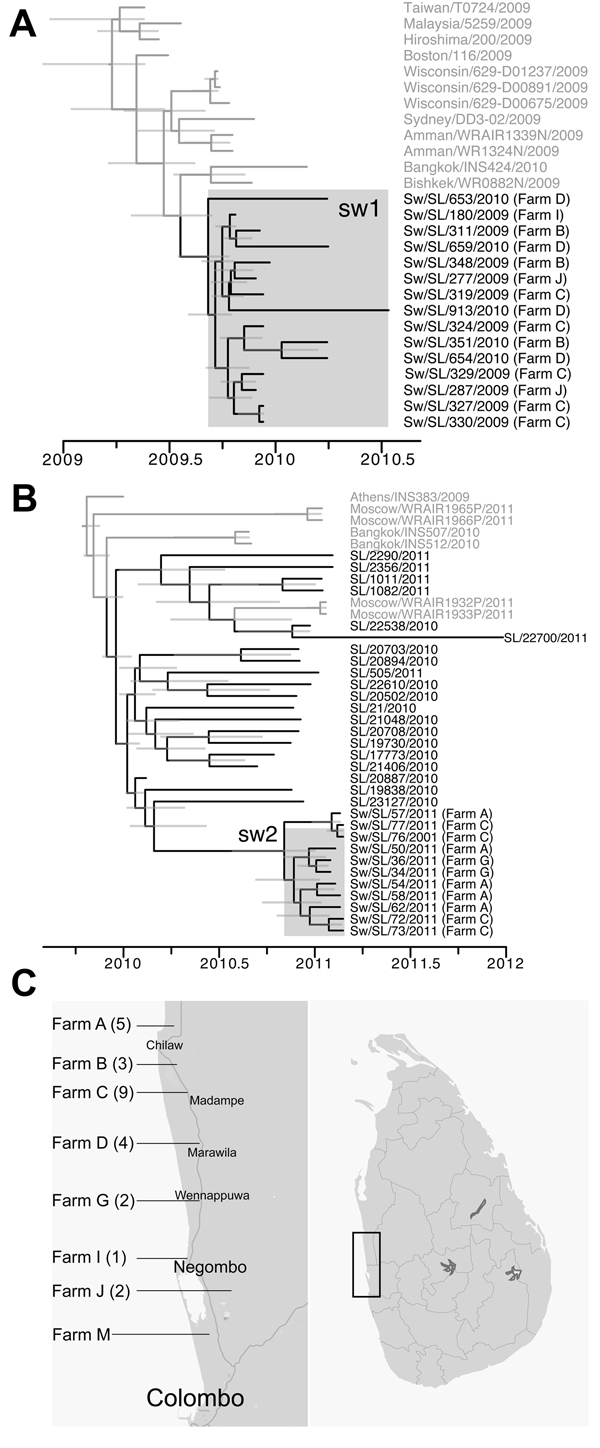
Phylogeny and divergence times of the concatenated whole genome of 2 swine pandemic influenza A(H1N1) (H1N1pdm) virus clusters (sw 1 and 2) detected in Sri Lanka (A,B) and distribution of swine farms yielding H1N1pdm virus isolates during 2009�?"2013. Gray branches represent global H1N1pdm viruses and black branches represent human and swine H1N1pdm viruses isolated in this study (A, B). Farm of origin is provided for all swine isolates. Gray bars on the tree nodes represent 95% highest posterior density intervals of divergence times. The maximum clade credible tree is summarized from 2 runs of 20 million generations (after the removal of the first 10% as burnin), using the uncorrelated lognormal relaxed clock model, the SRD06 codon based nucleotide substitution model and an exponential coalescent population size, in BEAST ( *7* ). The number of swine H1N1pdm viruses isolated in the farm is provided in brackets (C). The farms from which H1N1pdm viruses were isolated in 2011 are farms A, C, and G. The common transportation truck is the property of the farm M owner. Approximate locations are given.

In Sri Lanka, swine H1N1pdm clusters sw1 (2009/10) and sw2 (2011) were genetically distinct from each other and from other swine viruses isolated globally, indicating 2 separate introductions to local swine that circulated among swine for 11 and 4 months, respectively, for each cluster. The sw1 and sw2 lineages did not appear to establish long-term sustained transmission within pigs. However, the reduced surveillance of farms during the period 2012�?"2013 ( Technical Appendix Table 1) means that this conclusion has to be qualified in regard to the sw2 lineage. We did not identify ancestors of sw1 in Sri Lanka, however sw2 appears to have been directly derived from hu4, which included the majority of the human viruses (20/35) sequenced from Sri Lanka. The lack of identification of a human ancestor for sw1 may be related to insufficient human influenza genomic data obtained from Sri Lanka during 2009�?"2010 ( Figures 1 , 2 ). Although the 11 sw2 viruses were isolated from pigs on farms A, C, and G, which were separated by >25 km from each other ( Table , Figure 2 ), they form a monophyletic clade with no human isolates within this cluster. Even though the paucity of human viruses sampled is a limitation in this study, the data suggest a single introduction of human viruses into swine followed by transmission within and between swine farms for >4 months ( Figure 2 ).

**Table:** Demographic data, internal and external biosecurity measures practice by swine farms, Sri Lanka, 2009�?"2013*

Farm	Pig replacement source	Used specific clothing and footwear	Used disinfectants for wheel and foot baths	Dedicated employees for fattening and nursery units	Type of truck used to transport pigs to the GSHD	Visitor restriction grade	Service provider visits	Cats and/or dogs allowed in the facility	H1N1pdm detection
A	Internal	No	Yes	Yes	Hired/farm M	M	Nil	Yes	Clade 2
B	Internal	Yes	Yes	Yes	Dedicated	A	No	Yes	Clade 1
C	Internal	No	No	No	Hired/farm M	N	Occasionally	Yes	Clades1/2
D	Internal	No	No	Yes	Dedicated	M	Occasionally	Yes	Nil
E	Internal	No	No	No	Hired/farm M	N	Occasionally	Yes	Nil
F	Internal	No	No	No	Hired/farm M	M	Nil	Yes	Nil
G	Internal	No	No	No	Hired/farm M	M	Nil	Yes	Clade 2
H	Internal	No	No	No	Hired/farm M	M	Nil	Yes	Nil
I	Internal	No	No	No	Hired/farm M	M	Occasionally	Yes	Clade 1
J	Internal	No	No	No	Hired/farm M	N	Occasionally	Yes	Clade 1
K	Internal	No	No	No	Hired/farm M	M	Nil	Yes	Nil
L	ND	No	ND	ND	Hired/farm M	ND	ND	ND	Nil
M	External	No	No	No	Self-owned	M	Yes	Yes	Nil
N	Internal	No	No	No	Hired/farm M	N	Nil	Yes	Nil
O�?	NA	No	No	No	NA	N	Nil	Yes	Nil
P	Internal	No	No	No	Hired/farm M	N	Nil	Yes	Nil
Q�?�	Internal	Yes	No	Yes	NA	A	Yes	Yes	Nil
R	Internal	No	No	No	Hired/farm M	M	Occasionally	Yes	Nil
S	Internal	No	No	No	Hired/farm M	N	Occasionally	Yes	Nil

*None of the swine farms housed domestic poultry in the facility. Farms are listed in alphabetical order, A being in the northernmost location and S the southernmost farm in this study. GSHD, Government Slaughter House Dematagoda. The common transportation truck is the property of the farm M owner. Visitor restriction is scored as M-moderate, and A-absolute restriction, N-nil, on the basis of level of visitor restrictions (absolute/moderate/nil) and selection of veterinary surgeons (designated/freelance); service providers are veterinary surgeons, drug, and vaccine providers. NA, not applicable; ND, no data available.�?"�? Backyard slaughterhouse.�?"�?�Pigs not slaughtered at the GSHD.

To clarify transmission patterns between affected swine farms in Sri Lanka, we obtained contact patterns by interviewing pig farmers using a structured questionnaire ( Technical Appendix ) with approval from the Ethics Review Committee, Faculty of Medicine, Ragama, Sri Lanka. There was no evidence of movement of persons or fomites between farms. However, during the peak demand period (November�?"December) of each year that surveillance was performed, a common truck owned by farm M ( Table ), driven by a single driver and an assistant, provided transportation from multiple farms to the abattoir, including from affected farms A, C, and G ( Table ). On some occasions, animals taken to the abattoir for slaughter were returned to the farm. Pools of water or body fluids were often noted within this truck, and it is possible that viable swine influenza viruses may have survived for varying periods. We did not test these fluids from the common transportation truck for influenza viruses; this is also a limitation of the study. 

Of the 15 farms on which the common truck was used, swine on 3 (20%) were infected by a sw2 clade virus; on 2 farms on which the common truck was not used, no swine were infected. This association was not statistically significant (p = 1.0), however, given the small numbers of farms investigated. Our findings are consistent with dispersal of sw2 clade viruses in association with the truck to infect multiple farms that were geographically distant, but this remains unproven. It was previously documented that influenza viruses can remain viable for prolonged periods of time in water at a temperature of �%^28A�C (comparable ambient temperature in the Western Province, Sri Lanka) ( *10* ) and are reported to survive longer periods on nonporous surfaces ( *11* ). Influenza virus has been detected in air samples from rooms of experimentally infected pigs ( *12* ) and in the exhaust air samples collected up to 1 mile away from the index farm ( *13* ), indicating the possibility of aerosol transmission for some distance. Notably, studies of the swine populations in the United States have demonstrated spatial dissemination of swine influenza viruses of human origin to match long-distance swine movements ( *14* ).

Despite widespread inter-farm transmission of sw1 and sw2, our results show that only animals on farm C were infected in both spillover events. Farms A and G, on which swine were infected by sw2 in 2011, appeared not to have had infected swine during 2009�?"2010, as shown by both virus isolation and serologic testing ( Technical Appendix Table 1). Maternally derived antibodies transferred through colostrum from dams infected during 2009�?"2011 may have provided passive protection to offspring born in 2010�?"2011. On swine farms in Sri Lanka, female swine are used for breeding for �%^2�?"3 years. In experimental challenge, maternally derived antibodies provided some protection against disease, but not complete protection from infection ( *15* ).

## Conclusions

This study demonstrates natural independent spillover events of H1N1pdm influenza viruses from humans to swine. H1N1pdm viruses appear to be spread by multiple, discrete introductions to swine, after which clonal expansion occurs within the swine. The spread of such virus lineages across multiple farms is consistent with virus dispersal by breaches of external biosecurity measures, including the manner of swine transportation, although this remains unproven given the small sample size. Unlike classical swine influenza, North American triple reassortant, and European avian swine viruses that have persistently circulated among swine for several decades in other countries ( *15* ), H1N1pdm does not appear to establish long-term lineages in swine in the absence of further reassortment. This observation requires confirmation in other geographic settings.

Technical Appendix Overview of the swine industry in Sri Lanka and surveillance of human and swine pandemic influenza A(H5N1) viruses.

## References

[1] Vijaykrishna D , Poon LL , Zhu HC , Ma SK , Li OT , Cheung CL , Reassortment of pandemic H1N1/2009 influenza A virus in swine. Science . 2010 ; 328 : 1529 . 10.1126/science.1189132 20558710 PMC3569847

[2] Perera HK , Wickramasinghe G , Cheung CL , Nishiura H , Smith DK , Poon LL , Swine influenza in Sri Lanka. Emerg Infect Dis . 2013 ; 19 : 481 �?" 4 . 10.3201/eid1903.120945 23621918 PMC3647653

[3] Pond SLK , Frost SDW , Muse SV . HyPhy: hypothesis testing using phylogenies. Bioinformatics . 2005 ; 21 : 676 �?" 9 . 10.1093/bioinformatics/bti079 15509596

[4] Edgar RC . MUSCLE: A multiple sequence alignment method with reduced time and space complexity. BMC Bioinformatics . 2004 ; 5 : 113 . 10.1186/1471-2105-5-113 15318951 PMC517706

[5] Posada D . jModelTest: phylogenetic model averaging. Mol Biol Evol . 2008 ; 25 : 1253 �?" 6 . 10.1093/molbev/msn083 18397919

[6] Stamatakis A . RAxML-VI-HPC: maximum likelihood-based phylogenetic analyses with thousands of taxa and mixed models. Bioinformatics . 2006 ; 22 : 2688 �?" 90 . 10.1093/bioinformatics/btl446 16928733

[7] Drummond AJ , Suchard MA , Xie D , Rambaut A . Bayesian phylogenetics with BEAUti and the BEAST 1.7. Mol Biol Evol . 2012 ; 29 : 1969 �?" 73 . 10.1093/molbev/mss075 22367748 PMC3408070

[8] Baillie GJ , Galiano M , Agapow PM , Myers R , Chiam R , Gall A , Evolutionary dynamics of local pandemic H1N1/2009 influenza virus lineages revealed by whole-genome analysis. J Virol . 2012 ; 86 : 11 �?" 8 . 10.1128/JVI.05347-11 22013031 PMC3255882

[9] Sharma S , Joshi G , Dash PK , Thomas M , Athmaram TN , Kumar JS , Molecular epidemiology and complete genome characterization of H1N1pdm virus from India. PLoS ONE . 2013 ; 8 : e56364 . 10.1371/journal.pone.0056364 23457559 PMC3574146

[10] Stallknecht DE , Kearney MT , Shane SM , Zwank PJ . Effects of pH, temperature, and salinity on persistence of avian influenza viruses in water. Avian Dis . 1990 ; 34 : 412 �?" 8 . 10.2307/1591429 2142421

[11] Bean B , Moore BM , Sterner B , Peterson LR , Gerding DN , Balfour HH Jr . Survival of influenza viruses on environmental surfaces. J Infect Dis . 1982 ; 146 : 47 �?" 51 . 10.1093/infdis/146.1.47 6282993

[12] . Corzo CA , Romagosa A , Dee S , Gramer M , Morrison RB , Torremorell M . Relationship between airborne detection of influenza A virus and the number of infected. Vet J. 2013 ; 196 : 171 �?" 175 . 10.1016/j.vaccine.2010.12.096 23164957 PMC3582798

[13] Torremorell M , Allerson M , Corzo C , Diaz A , Gramer M . Transmission of Influenza A Virus in Pigs. Transbound Emerg Dis . 2012 ; 59 ( Suppls1 ): 68 �?" 84 . 10.1111/j.1865-1682.2011.01300.x 22226050

[14] Nelson MI , Lemey P , Tan Y , Vincent A , Lam TT , Detmer S , Spatial dynamics of human-origin H1 influenza A virus in North American swine. PLoS Pathog . 2011 ; 7 : e1002077 . 10.1371/journal.ppat.1002077 21695237 PMC3111536

[15] Loeffen WL , Heinen PP , Bianchi AT , Hunneman WA , Verheijden JH . Effect of maternally derived antibodies on the clinical signs and immune response in pigs after primary and secondary infection with an influenza H1N1 virus. Vet Immunol Immunopathol . 2003 ; 92 : 23 �?" 35 . 10.1016/S0165-2427(03)00019-9 12628761

