# Supplementary material for: Molecular Epidemiology of Influenza A(H1N1)pdm09 Virus among Humans and Swine, Sri Lanka
Source: Emerg Infect Dis. 2014 Dec;20(12):2080–4. doi: 10.3201/eid2012.140842 (PMC4257816; doi:10.3201/eid2012.140842)
Supplement: Technical Appendix — Overview of the swine industry in Sri Lanka and surveillance of human and swine pandemic influenza A(H5N1) viruses. [file 14-0842-Techapp-s1.pdf]

DOI: http://dx.doi.org/10.3201/eid.2012.140842

Molecular Epidemiology of A(H1N1)pdm09 Virus among Humans and Swine, Sri Lanka

Technical Appendix: Overview of the swine industry and related influenza surveillance in Sri Lanka

Swine farms in Sri Lanka are predominantly (~61%) located in the western coastal belt spanning Puttlam, Gampaha, Colombo and Kalutara administrative districts of the country, which is identified as the 'pig belt" of Sri Lanka, with a standing population of approx. 80,000 pigs. Pigs are not imported on a regular basis to the country (*1*).

The mean slaughter age and average live weight of a local pig is approx. 9.8 months (4.4-10.1 months) and about 84 kg, respectively (*2*). However, the mean age of the pigs included in the current study was 6.9 months (and ranged from 6.0–9.0 months).

Pigs were transported in completely covered box-type trucks, whose upper half of rear door is kept open during transportation. The average travel time that animals experience was 3–4 hours, with the exception of farm H that took 8–10 hours (Table, Technical Appendix Table 1, 2). Animals are not pooled within the same truck during a single transportation session.

The Government Slaughterhouse located in Dematagoda, Colombo, which operates for 6 days per week and slaughters around 20 pigs per day or depending on the available number of pigs on a given day. More than 90% of the animals that are slaughtered at the Dematagoda Government Swine Slaughterhouse receive pigs from the swine farms located in the Puttlam, Gampaha, Colombo and Kalutara administrative districts. Routine vaccination against influenza on both human and swine is not carried in the country. However, a single vaccination program against H1N1pdm was conducted in late 2010 on humans (*3*). Nevertheless, none of the farmers or employees of the swine farms had received vaccination.

Swine Surveillance

Swine swab samples were obtained from freshly slaughtered pigs at the Government Slaughterhouse Dematagoda Colombo, from August 2009 to March 2013. Swab (tracheal and nasal) and serum samples were collected from these pigs on weekly basis from otherwise healthy animals (Technical Appendix Figure). The swine swabs samples were inoculated into Madin-Darby canine kidney (MDCK) monolayers and 9-11days old embryonated chicken eggs through allantoic route. One blind passage was performed on each negative swine swab sample. Hemagglutination inhibition assay was performed using panel of viral antigens stated in our previous publication (*4*).

Molecular Detection of Human H1N1pdm Viruses

Influenza A/B typing panel and CDC real time qRT-PCR (A/H1/H3/H1N1pdm09) subtyping panel were used for screening and detection. In summary, viral RNA were extracted using QIAamp™ Viral RNA Mini kits, and 5.5 µL nuclease free water, 0.5 µL of each forward primer and reverse primer, 0.5 µL probe, 0.5 µL superscript TM III/RT-Platinum, 12.5 µL 2 x PCR buffer were run in each qRT- PCR in accordance with following conditions; reverse Transcript 50°C x 30 min, Taq inhibitor activation 95°C x 2min, PCR amplification (40 cycles); 95°C x15sec min and 55°C x 30 sec and fluorescence data was obtained as described in the protocol (*5*). Human samples tested positive for H1N1pdm viruses were inoculated into MDCK cells as described elsewhere (*4*) and two blind passages were performed on each culture negative sample.

Temporal Phylogenetic Analysis

Bayesian Markov chain Monte Carlo analyses were performed using the SRD06 codon based nucleotide substitution model (*6*), a flexible coalescent based demographic model, and the Gaussian Markov Random Field model (*7*). These models were selected as these have been consistently been shown to be the best-fit models for influenza viruses (*8–10*).

References

1. Abeyrthne AS. A review of the livestock industry of Sri Lanka. Kandy (Sri Lanka): Kandy Printers; 2007. p. 57–65.

2. Gajanayake S. Swine Industry in Western Province: II. Feeding Practices. Sri Lanka Veterinary Journal. 2004;51(A1):11–4. http://journal.slva.org/content/journal/id/15

3. Ministry of Healthcare and Nutrition, Epidemiology Unit, Sri Lanka. Organization of vaccination for individuals with high risk of severe disease and complications of pandemic influenza A/H1N1. Nov. 2010. http://www.epid.gov.lk/web/attachments/article/180/Vaccinations%20for%20individuals.pdf [cited 2014 May 01].

4. Perera HK, Wickramasinghe G, Cheung CL, Nishiura H, Smith DK, Poon LL, et al. Swine influenza in Sri Lanka. Emerg Infect Dis. 2013;19:481–4. PubMed http://dx.doi.org/10.3201/eid1903.120945

5. WHO Collaborating Centre for Influenza, CDC Atlanta, GA, USA. http://www.who.int/csr/resources/publications/swineflu/CDCrealtimeRTPCRprotocol_20090428.pdf [cited 2014 May 01].

6. Minin VN, Bloomquist EW, Suchard MA. Smooth skyride through a rough skyline: Bayesian coalescent-based inference of population dyamics. Mol Biol Evol. 2008;25:1459–71. PubMed http://dx.doi.org/10.1093/molbev/msn090

7. Shapiro B, Rambaut A, Drummond AJ. Choosing appropriate substitution models for the phylogenetic analysis of protein-coding sequences. Mol Biol Evol. 2006;23:7–9. PubMed http://dx.doi.org/10.1093/molbev/msj021

8. Drummond AJ, Suchard MA, Xie D, Rambaut A. Bayesian phylogenetics with BEAUti and the BEAST 1.7. Mol Biol Evol. 2012;29:1969–73. PubMed http://dx.doi.org/10.1093/molbev/mss075

9. Rambaut A, Pybus OG, Nelson MI, Viboud C, Taubenberger JK, Holmes EC. The genomic and epidemiological dynamics of human influenza A virus. Nature. 2008;453:615–9. PubMed http://dx.doi.org/10.1038/nature06945

10. Vijaykrishna D, Smith GJ, Pybus OG, Zhu H, Bhatt S, Poon LL, et al. Long-term evolution and transmission dynamics of swine influenza A virus. Nature. 2011;473:519–22. PubMed http://dx.doi.org/10.1038/nature10004

Technical Appendix Table 1. Swine swab and serum samples tested for pandemic influenza A(H1N1), Sri Lanka*

Farm	2009	2010		2011		2012		2013	
	Virus isolation (%)	Virus isolation (%)	Sero positive (%)	Virus isolation (%)	Sero positive (%)	Virus isolation (%)	Sero positive (%)	Virus isolation (%)	Sero positive (%)
A	0/22	0/47	0/10 (0)	5/49 (10.2)	13/49 (26.5)	0/60	0/60 (0)	0/11	1/11 (9.1)
B	3/81 (3.7)	0/256	5/30 (16.6)	0/343	72/343 (20.9)	0/423	12/423 (2.8)	0/318	87/318 (27.3)
C	5/81 (6.1)	0/25	7/16 (43.7)	4/76 (5.2)	22/76 (28.9)	NC	NC	NC	NC
D	0/22	4/180 (2.2)	4/49 (8.1)	0/156	46/156 (29.4)	0/178	2/178 (1.1)	0/105	12/105 (11.4)
E	NC	0/40	0/20 (0)	NC	NC	NC	NC	NC	NC
F	NC	NC	NC	0/3	0/32 (0)	NC	NC	NC	NC
G	NC	0/38	NC	2/47 (4.2)	19/47 (40.4)	NC	NC	NC	NC
H	NC	0/29	NC	NC	0/5(0)	0/37	0/37 (0)	NC	NC
I	1/68 (1.4)	0/166	4/28 (14.2)	0/164	53/164 (32.3)	0/172	1/172 (0.6)	0/145	24/145 (16.5)
J	2/24	0/44	NC	NC	NC	NC	NC	NC	NC
K	0/37	0/67	5/28 (17.8)	NC	NC	NC	NC	NC	NC
L	NC	0/28	0/10 (0)	NC	NC	NC	NC	NC	NC
M	NC	0/95	1/13 (17.8)	0/52	16/52 (30.7)	0/241	16/241 (6.6)	0/269	51/269 (18.9)
N	NC	NC	NC	NC	NC	0/5	0/5(0)	NC	NC
O	NC	NC	NC	0/21	0/21 (0)	NC	NC	NC	NC
P	NC	0/41	0/18 (0)	NC	NC	NC	NC	NC	NC
Q	NC	NC	NC	0/17	2/17 (11.7)	NC	NC	NC	NC
R	NC	0/94	5/29 (7.6)	NC	NC	NC	NC	NC	NC
S	0/21	0/43	0/5 (0)	0/61	31/61 (50.8)	0/129	3/129 (2.3)	NC	NC

*Farms are listed in alphabetical order, A being in the northernmost location and S the southernmost farm in this study.

Technical Appendix Table 2. Herd size, replacement sources and replacement frequency of swine farms, Sri Lanka

Farm	Approximate herd size	Swine replacement source	Replacement frequency of animals per month
A	200	Internal	30
B	1,500	Internal	30
C	400	Internal	20
D	750	Internal	30
E	250	Internal	10
F	100	Internal	10
G	200	Internal	10
H	100	Internal	10
I	500	Internal	40
J	400	Internal	15

Farm	Approximate herd size	Swine replacement source	Replacement frequency of animals per month
K	300	Internal	15
L	Data not available	Data not available	Data not available
M	300	External	30
N	50	Internal	5
O	Inapplicable	Inapplicable	Inapplicable
P	100	Internal	10
Q	750	Internal	75
R	500	Internal	10
S	160	Internal	10

*Farms are listed in alphabetical order, A being in the northernmost location and S the southernmost farm in this study.

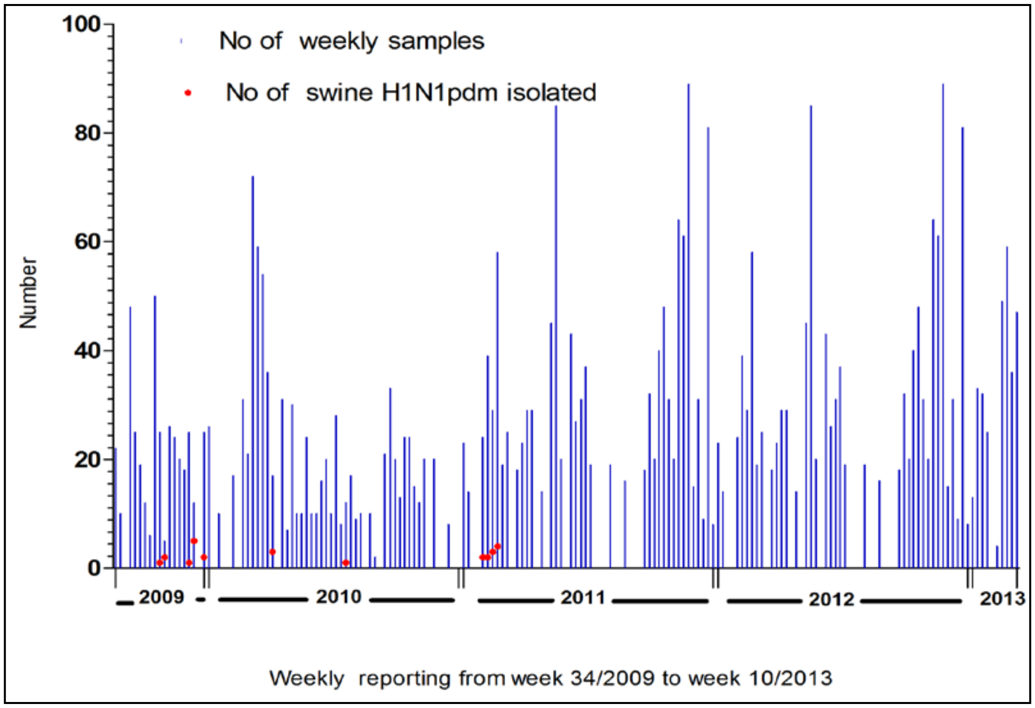

Technical Appendix Figure. Weekly sample distribution. Weekly numbers of pigs sampled from week 34/2009 to week 10/2013. Blue bars indicate the number of pigs sampled each week. Red dots indicate number of swine influenza viruses isolated.

Swine Farm Surveillance Questionnaire:

a) **General characteristics**

1) Identity of the farm……………………………………………………………………

2) Address of the farm………………………………………….......................……….

3) Contact Tel No …………………………………

4) Herd size……………

5) No of fattening units……………………………

6) Replacements source/s

   a) Internal ☐

   b) External ☐

   c) Both ☐

   d)

   e) If answered to b or c, the source/s

   1……………………………………………………………………………………..

   2…………………………………………………………………………………….

   3……………………………………………………………………………………..

   4…………………………………………………………………………………….

7) Replacement frequency

   a) …………

8) Does the farm sell surplus piglets to other farms

   a) Yes ☐

   b) No ☐

c) If answered yes, name and the address of the farm/s

1……………………………………………………………………………………
2……………………………………………………………………………………
3…………………………………………………………………………………….

9) Type of separation between pens:

a) Solid walls ☐

b) Bars ☐

c) other ………………..

10) Pen stocking density (m^2)……………………………..

11) Length of the fattening period………………………..

12) No of workers in the farm:

a) Full-time ………
b) Casual…………

13) Educational level of the farmer:

a) No Formal education ☐

b) Primary ☐

c) Secondary ☐

d) University degree ☐

14) Obtained further training on pig framing:

a) Yes ☐

b) No ☐

15) Aware that human diseases could transmit to pigs and *vice versa*:

a) Yes ☐

b) No ☐

b) **Farm biosecurity**

1) Availability of outside fence:

   a) Yes ☐

   b) No ☐

2) Pigs are allowed to move out the pens:

   a) Yes ☐

   b) Occasionally ☐

   c) Never ☐

3) Use of sanitising wheel baths:

   a) Always ☐

   b) Occasionally ☐

   c) Never ☐

4) Generic name/s of the chemical used in wheel bath
   1………………………….2……………………..

5) Use of sanitising boot baths:

   a) Always ☐

   b) Occasionally ☐

   c) Never ☐

6) Generic name/s of the chemical used in boot bath
   1………………………….2……………………..

7) Replacement frequency of wheel/boot bath solution:

   a) 12hr ☐

   b) 24hr ☐

   c) 36hr ☐

e) 48hr ☐

f) 1/52 ☐

8) Separate foot bath for each pen:

a) Yes ☐

b) No ☐

9) Wearing of dedicated clothes before entering the facility:

a) Always ☐

b) Occasionally ☐

c) Never ☐

10) Presence of changing rooms and shower in the farm:

a) Available ☐

b) Not available ☐

11) Having a shower before entering the facility:

a) Always ☐

b) Occasionally ☐

c) Never ☐

12) Restrictions on entering workers suffering from flu like symptoms in to the farm

a) Always ☐

b) Occasionally ☐

c) Never ☐

13) Sharing workers with other swine farms:

a) Always ☐

b) Occasionally ☐

c) Never ☐

If answered to a or b, name of the farm/s

1………………………………………………

2………………………………………………

3……………………………………………….

14) Do workers employed in the fattening unit work in the nursery unit:

a) Yes ☐

b) No ☐

15) How are the farm animals transported to the slaughterhouse

a) Dedicated vehicle ☐

b) Common vehicle in the farm ☐

c) Hired vehicle ☐

If answered to c, does this vehicle provide service to other swine farms?

a) Yes ☐

b) No ☐

If answered yes, name the farm/s

1…………………………………………………………………

2…………………………………………………………………

3…………………………………………………………………

16) Vehicle use to transport nursery pigs to fattening pens

a) Dedicated vehicle belongs to the farm ☐

b) Common vehicle in the farm ☐

c) Hired vehicle ☐

c) If answered to c, the source/s of the vehicle

1……..............................2………………………………

17) Are animals sourced from different farms “pooled “ during transportation to the slaughterhouse:

a) Always ☐

b) Occasionally ☐

c) Never ☐

c) If answered to b or c, name and the address of the farm/s

1…………………………………………………………………………………
2…………………………………………………………………………………
3…………………………………………………………………………………
4…………………………………………………………………………………

19). Presence of other domesticated animals in the farm

a) Always ☐

b) Occasionally ☐

c) Never ☐

d) Type of animal/s (if answered yes to a or b)………/……………./………….

c) **Feeding management**

1) Animals are feed:

a) Manually ☐

b) Automatically ☐

2) Type of feed:

a) Swill ☐

b) Rice barn ☐

c) Kitchen refuse ☐

d) Non-human edible chicken refuses☐

3) Source of food:

a) Self made ☐

b) Brought from one source ☐

c) Different sources ☐

d) If answered to c and d, address/es of the feeding source

1…………………………………………………………………………………………

2…………………………………………………………………………………………

3…………………………………………………………………………………………

4……………………………………………………………………………………....

d) **Visitor restrictions**

1. Restrictions on people visiting the farm:

a) Always ☐

b) Occasionally ☐

c) Never ☐

2. Do service providers * visit the farm

a) Always ☐

b) Occasionally ☐

c) Never ☐

d) If answered to b or c, indicate the purpose/es and identity of the service provider

1 name ………………………service 1…………………2…………………3…………..

2 name ………………………service 1…………………2…………………3…………..

3 name ………………………service 1…………………2…………………3…………..

4 name ………………………service 1…………………2…………………3…………..

5 name ………………………service 1…………………2…………………3…………..

3. How often people stated above visit the farm

a) Weekly ☐

b) Monthly ☐

c) > one month ☐

4. Does the farm obtain vet surgeon's consultations

    a) Yes ☐

    b) No ☐

    if yes name of the vet surgeon/s

    1.……………………………………………….

    2. ……………………………………………….

5. Does the farm obtain consultations from one designated vet surgeon, when animal/s fall sick:

a) The farm has selected dedicated vet surgeon ☐

b) Not confined to one vet surgeon ☐

6. How often designated "farm" vet visit the premises

a) Weekly ☐

b) Monthly ☐

c) > one month ☐

**Service providers: Sales representatives, drug suppliers, vaccine suppliers, feed suppliers, artificial insemination providers…etc.*
